# Learning mixed graphical models with separate sparsity parameters and stability-based model selection

**DOI:** 10.1186/s12859-016-1039-0

**Published:** 2016-06-06

**Authors:** Andrew J. Sedgewick, Ivy Shi, Rory M. Donovan, Panayiotis V. Benos

**Affiliations:** Department of Computational and Systems Biology, University of Pittsburgh School of Medicine, Pittsburgh, PA USA; Joint Carnegie Mellon University-University of Pittsburgh PhD Program in Computational Biology, Pittsburgh, PA USA; Department of Bioengineering, University of Pittsburgh, Pittsburgh, PA USA

## Abstract

**Background:**

Mixed graphical models (MGMs) are graphical models learned over a combination of continuous and discrete variables. Mixed variable types are common in biomedical datasets. MGMs consist of a parameterized joint probability density, which implies a network structure over these heterogeneous variables. The network structure reveals direct associations between the variables and the joint probability density allows one to ask arbitrary probabilistic questions on the data. This information can be used for feature selection, classification and other important tasks.

**Results:**

We studied the properties of MGM learning and applications of MGMs to high-dimensional data (biological and simulated). Our results show that MGMs reliably uncover the underlying graph structure, and when used for classification, their performance is comparable to popular discriminative methods (lasso regression and support vector machines). We also show that imposing separate sparsity penalties for edges connecting different types of variables significantly improves edge recovery performance. To choose these sparsity parameters, we propose a new efficient model selection method, named Stable Edge-specific Penalty Selection (StEPS). StEPS is an expansion of an earlier method, StARS, to mixed variable types. In terms of edge recovery, StEPS selected MGMs outperform those models selected using standard techniques, including AIC, BIC and cross-validation. In addition, we use a heuristic search that is linear in size of the sparsity value search space as opposed to the cubic grid search required by other model selection methods. We applied our method to clinical and mRNA expression data from the Lung Genomics Research Consortium (LGRC) and the learned MGM correctly recovered connections between the diagnosis of obstructive or interstitial lung disease, two diagnostic breathing tests, and cigarette smoking history. Our model also suggested biologically relevant mRNA markers that are linked to these three clinical variables.

**Conclusions:**

MGMs are able to accurately recover dependencies between sets of continuous and discrete variables in both simulated and biomedical datasets. Separation of sparsity penalties by edge type is essential for accurate network edge recovery. Furthermore, our stability based method for model selection determines sparsity parameters faster and more accurately (in terms of edge recovery) than other model selection methods. With the ongoing availability of comprehensive clinical and biomedical datasets, MGMs are expected to become a valuable tool for investigating disease mechanisms and answering an array of critical healthcare questions.

## Background

Integrating biomedical datasets from different data streams (e.g., omics, clinical) and of different types (continuous, discrete) is of utmost importance and has become an analysis bottleneck in biomedical research. Ideally, one would like to be able to uncover all direct associations between variables and/or perform feature selection and classification tasks using all data. The first task can reveal disease mechanisms and the second can be used to select variables characteristic of disease status, therapy outcome or any other variable of clinical importance. Graphical models have been used in the past for both of these tasks, but they are often limited to datasets with discrete-only or continuous-only variables. Traditional univariate approaches for feature selection exist as well, but they also often operate on a single data type. In addition, due to the high dimensionality and co-linearity of biological data, markers selected by these standard feature selection algorithms can be unstable and lack biological relevance [2], a problem that has recently been addressed directly [3]. Many existing models that do integrate different data types make heavy use of prior knowledge [4, 5] and as such are not easily extendable to clinical and other data that are not well studied. As a result, although numerous biomedical data sets exist with genomic, transcriptomic and epigenetic data for each sample, a general framework for integrative analysis of these heterogeneous data is lacking.

In this paper, we study several strategies for learning the structure of graphical models over mixed data types (discrete and continuous) to produce statistically and biologically meaningful predictive models. We measure the performance of these strategies in synthetic data (via true edge recovery) and biological data (via functional enrichment and performance on classification tasks).

The major contributions of this paper are threefold. First, we apply an MGM, proposed by Lee and Hastie [6], to simulated and biological datasets. These datasets have higher dimensionality and are derived from more complicated network structures than datasets used in previous work with this model. Second, we propose the use of a separate sparsity penalty for each edge type in the MGM, which significantly improves performance. Third, to assist with setting the sparsity parameters we use a heuristic search based on an existing model selection method (stability approach to regularization selection – StARS) [1], that outperforms standard methods.

### Prior work

Graphical models are a natural tool for decoding the complex structure of heterogeneous data and allow for integration of many data types. They learn a network of statistical dependencies subject to a joint probability distribution over the data. Mixed graphical models (MGMs) are graphical models learned over a mixture of continuous and discrete features.

A fully specified conditional Gaussian MGM, as characterized by Lauritzen & Wermuth [7] 26 years ago, would require different continuous distribution parameters for every possible setting of the discrete variables. Restricting ourselves to “homogeneous” models, which use a common covariance matrix for continuous variables independent of the discrete variable values, is therefore necessary to avoid trying to learn a parameter space that is exponential in the number of variables. Similar to pairwise Markov Random Fields over only discrete variables, the main hurdle to the calculation of likelihood in MGMs is calculation of the partition function. This computation is intractable with a large number of discrete variables because it requires summing over all possible discrete variable settings. Two approaches to get around this partition function calculation are: (1) learn separate regressions of each variable given all of the others [8–10], and (2) maximize a tractable pseudolikelihood function instead of the actual likelihood [6].

Performing separate regressions is a common approach to the MGM learning problem. This class of methods learns a conditional distribution for each node given the rest. Examples of this strategy include estimation of the sparse inverse covariance matrix of a multivariate Gaussian by Meinshausen and Bühlmann [11], and estimation of mixed variable networks via random forests [8] or exponential families [9, 10]. Alternatively, the pseudolikelihood, proposed by Brasg [12], is a consistent estimator of the likelihood, and is defined as the product of the conditional distributions of each node given the rest. Both of these approaches thus avoid calculation of the partition function for the joint distribution by substituting the conditional distributions of each node into the optimization problem. Separate regressions offer flexibility and are easily parallelized, but in both the continuous [13] and mixed cases [6] estimating the parameters by maximizing the likelihood or pseudolikelihood, respectively, has the advantage of better empirical performance. Because of this we chose the focus our efforts on the MGM learning approach via pseudolikelihood, as proposed in Lee and Hastie [6].

Although Lee and Hastie do not test their algorithm on high-dimensional data, we find that their model is well suited for high-dimensional learning due to their inclusion of a sparsity penalty on the parameters. An important issue that we ran into in our experiments was that the model would often select too many continuous-continuous edges and too few edges involving discrete variables. This is likely a combination of the phenomenon observed in [9] where linear regressions have better edge prediction performance than logistic regression between the same nodes and the fact that Lee and Hastie use the same sparsity penalty on all edges regardless of the type(s) of nodes they connect. Lee and Hastie use a weighting scheme to take into account discrete variables with differing numbers of categories, but this does not solve this problem. Therefore, in this paper we introduce a new regularization method for the Lee and Hastie’s model that uses a different penalty for each type of edge: continuous-continuous, continuous-discrete, and discrete-discrete. In addition, because this approach creates more parameters for the user to set, we present an edge stability based method for selecting the three sparsity parameters. We call the combination of using separate sparsity penalties with our heuristic search Stable Edge-specific Penalty Selection (StEPS).

## Methods

### Mixed graphical models

Lee and Hastie [6] parameterize a mixed graphical model over p Gaussian variables, *x*, and q categorical variables, *y*, as a pairwise Markov Random Field. Here we briefly summarize their model:

$$ p\left(x,\ y,\ \varTheta \right)\propto \exp \left({\displaystyle \sum_{s=1}^p}{\displaystyle \sum_{t=1}^p}-\frac{1}{2}{\beta}_{st}{x}_s{x}_t+{\displaystyle \sum_{s=1}^p}{\alpha}_s{x}_s+{\displaystyle \sum_{s=1}^p}{\displaystyle \sum_{j=1}^q}{\rho}_{sj}\left({y}_j\right){x}_s+{\displaystyle \sum_{j=1}^q}{\displaystyle \sum_{r=1}^q}{\phi}_{rj}\left({y}_r,{y}_j\right)\right) $$

In this model *β*_*st*_ represents the interaction between two continuous variables, *x*_*s*_ and *x*_*t,*_*ρ*_*sj*_(*y*_*j*_) is a vector of parameters that correspond to the interaction between the continuous variable *x*_*s*_ and the categorical variable *y*_*j*_ indexed by the levels (i.e., categories) of the variable *y*_*j*_, and *ϕ*_*rj*_(*y*_*r*_, *y*_*j*_), is a matrix of parameters indexed by the levels of the categorical variables *y*_*j*_, and *y*_*r*_. In the continuous only case, this model reduces to a multivariate Gaussian model where the *β*_*st*_ parameters are entries in the precision matrix. In the categorical only case, this model is the popular pairwise Markov random field with potentials given *ϕ*_*rj*_(*y*_*r*_, *y*_*j*_); and it could parameterize an Ising model as in the binary-only case, for example,. Thus the model serves as a generalization of two popular uni-modal models to the multi-modal regime.

In order to avoid the computational expense of calculating the partition function of this model, Lee and Hastie optimize the negative log pseudolikelihood, which is:

$$ \tilde{l}\left(\varTheta\;\Big|x,y\right) = -{\displaystyle \sum_{s=1}^p} \log\ p\left({x}_s\Big|{x}_{\backslash s},\ y;\varTheta \right) - {\displaystyle \sum_{r=1}^q} \log p\left({y}_r\Big|x,\ {y}_{\backslash r};\ \varTheta \right) $$

To ensure a sparse model, $\tilde{l}$ is minimized with respect to a sparsity penalty, *λ*:

$$ {\mathrm{minimize}}_{\Theta}\ \tilde{l}\left(\Theta \right) + \lambda \left({\displaystyle \sum_{t<s}}\left|{\beta}_{st}\right| + {\displaystyle \sum_{s,j}}{\left\Vert {\rho}_{sj}\right\Vert}_2 + {\displaystyle \sum_{r<j}}{\left\Vert {\phi}_{rj}\right\Vert}_F\right) $$

where Θ is a shorthand for all of the model parameters. The parameter matrices *β* and *ϕ* are symmetric, so only half of each matrix is penalized. Lee and Hastie use an accelerated proximal gradient method to solve this optimization problem.

A standard way of handling a categorical variable with L levels is to convert the variable to L-1 indicator variables where the last level is encoded by setting all indicators to zero, this is necessary to ensure the linear independence of variables in the regression problem. Lee and Hastie’s MGM approach, uses L indicator variables (i.e., the elements of *ρ*_*sj*_(*y*_*j*_) and *ϕ*_*rj*_(*y*_*r*_, *y*_*j*_)) to improve interpretability of the model, and enforces a group penalty to ensure the indicator coefficients sum to zero.

To perform our experiments we adapted the Matlab code provided by Lee and Hastie (available at http://www.eecs.berkeley.edu/~jasondlee88/learningmgm.html).

### Separate sparsity penalties

Our main modification to the Lee and Hastie model itself is that we use different sparsity penalties for the three edge types: edges connecting two continuous nodes (*cc*), edges connecting a continuous and discrete node (*cd*) and edges connecting two discrete nodes (*dd*). With these penalties, the new optimization problem becomes:

$$ {\mathrm{minimize}}_{\Theta}\ \tilde{l}\left(\Theta \right) + {\lambda}_{cc}{\displaystyle \sum_{t<s}}\left|{\beta}_{st}\right| + {\lambda}_{cd}{\displaystyle \sum_{s,j}}{\left\Vert {\rho}_{sj}\right\Vert}_2 + {\lambda}_{dd}{\displaystyle \sum_{r<j}}{\left\Vert {\phi}_{rj}\right\Vert}_F $$

### Methods for model selection

K-fold cross-validation (CV) [14] splits the data into K subsets and holds each set out once for validation while training on the rest. We use K = 5 and average the negative log-pseudolikelihood of the test sets given the trained models. The Akaike information criterion (AIC) [15] and Bayes information criterion (BIC) [16] are model selection methods that optimize the likelihood of a model based on a penalty on the size of the model represented by degrees of freedom. To calculate the AIC and BIC, we substitute the pseudolikelihood for the likelihood and we define the degrees of freedom of the learned network as follows.

In the standard lasso problem, the degrees of freedom is simply the number of non-zero regression coefficients [17]. So, in the continuous case, the degrees of freedom of a graphical lasso model is the number of edges in the learned network. In the mixed case, edges incident to discrete variables have additional coefficients corresponding to each level of the variable. Lee and Hastie’s MGM uses group penalties on the edge vectors, *ρ*, and matrices, *ϕ*, to ensure that all dimensions sum to zero. So, in the model, an edge between two continuous variables adds one degree of freedom, and edge between a continuous variable and a categorical variable with L levels adds L-1 degrees of freedom, and an edge between two discrete variables with L_i_ and L_j_ levels adds (L_i_ – 1)(L_j_ - 1) degrees of freedom.

We compare these model selection methods to an oracle selection method. For the oracle model, we select the sparsity parameters that minimize the number of false positives and false negatives between the estimated graph and the true graph. While we do not know the true graph in practice and none of the other methods use the true graph, this method shows us the best possible model selection performance under our experimental conditions.

AIC, BIC, and CV all require calculating the pseudolikelihood from a learned model so to optimize over separate sparsity penalties for each edge type, we perform a cubic grid search of *λ*_*cc*_, *λ*_*cd*_, and *λ*_*dd*_ over {.64, .32, .16, .08, .04}.

### Stability for model selection

Here we briefly present the StARS procedure [1] reformulated in terms of *λ* rather than *Λ* = 1/*λ* as was originally described. Given a dataset with *n* samples, StARS draws *N* subsamples of size *b* without replacement from the set of $$ \left(\begin{array}{c}\hfill n\hfill \\ {}\hfill b\hfill \end{array}\right) $$ possible subsamples. An MGM network is learned for each subsample over a user specified set of values and a single sparsity parameter, *λ*. The adjacency matrices from these learned models are used to calculate, ${\widehat{\theta}}_{st}\left(\lambda \right)$, the fraction of subsample networks that predict an edge from node *s* to node *t*. Using this value we can then calculate edge instability, ${\widehat{\xi}}_{st}\left(\lambda \right) = 2{\widehat{\theta}}_{st}\left(\lambda \right)\left(1 - {\widehat{\theta}}_{st}\left(\lambda \right)\right)$, which is the empirical probability of any two subsample graphs disagreeing on each possible edge at each value of *λ*. Liu et al. define total instability of the graph, $\widehat{D}\kern0.22em \left(\lambda \right)$, as the average of ${\widehat{\xi}}_{st}\left(\lambda \right)$ over all edges: $$ \widehat{D}\left(\lambda \right) = \frac{{\displaystyle {\sum}_{s<t}}{\widehat{\xi}}_{st}\left(\lambda \right)}{\left(\begin{array}{c}\hfill p+q\hfill \\ {}\hfill 2\hfill \end{array}\right)} $$. Very low values of *λ* will result in very dense but stable graph, which is not desirable. To avoid this, StARS monotonizes the instability: $\overline{D}\left(\lambda \right)={ \sup}_{\lambda \le t}\ \widehat{D}(t)$ and selects $\widehat{\lambda}= \inf \left\{\lambda\ :\ \overline{D}\left(\lambda \right)\le \gamma \right\}$ with *γ* being a user defined threshold (called *β* in [1]). In other words, starting with a large value of *λ* that produces an empty graph, we reduce *λ* until the total instability hits the given threshold.

### Stable Edge-specific Penalty Selection (StEPS)

We modified the StARS procedure to accommodate selection of separate *λ* for each edge type. We now define the total instability over each edge type instead of the entire graph: $$ {\widehat{D}}_{cc}\left(\lambda \right) = \frac{{\displaystyle {\sum}_{cc}}{\widehat{\xi}}_{st}\left(\lambda \right)}{\left(\begin{array}{c}\hfill p\hfill \\ {}\hfill 2\hfill \end{array}\right)} $$, ${\widehat{D}}_{cd}\left(\lambda \right) = \frac{{\displaystyle {\sum}_{cd}}{\widehat{\xi}}_{st}\left(\lambda \right)}{pq}$, $$ {\widehat{D}}_{dd}\left(\lambda \right) = \frac{{\displaystyle {\sum}_{dd}}{\widehat{\xi}}_{st}\left(\lambda \right)}{\left(\begin{array}{c}\hfill q\hfill \\ {}\hfill 2\hfill \end{array}\right)} $$. Given these separate estimates of total instability, we then perform the rest of the StARS algorithm for each *λ*. This approach does not require any additional model learning, the only extra computations in this approach compared to the standard, single penalty StARS are the additional averages, which are trivial to calculate. Because the subsample network learning uses the single penalty MGM, this procedure is linear in the size of the parameter search space. Based on the suggestions in [1], and the default parameters in the R implementation of StARS [18], we use $N=20,\ b=10\sqrt{n}$, and *γ* = .05.

### Simulated network data

We generated 20 scale-free networks of 100 variables each, based on the framework of Bollobás et al. [19] but ignoring edge direction. So, given a number of nodes to connect, we start with an edge between two nodes and the rest of the nodes unconnected, we iteratively add edges until all nodes are connected. At each edge addition, we connect two non-zero degree nodes with probability .3; and we connect a node *i* with degree 0 to a node *j* with non-zero degree with probability 0.7. In each case, the non-zero degree nodes are selected randomly with probability proportional to their degree: $\frac{\mathrm{degree}(j)}{{\displaystyle {\sum}_{k\ \in V}} degree(k)}$.

For each network we simulated two datasets of 500 samples with 50 continuous and 50 categorical variables. Each categorical variable had 4 levels. The parameters in one dataset were set so that discrete-continuous and discrete-discrete edges had approximately linear interactions, while the other dataset did not have this constraint. Each edge, from node *s* to node *t* is given a weight, *w*_*st*_, drawn uniformly from [.5, .8]. For continuous-continuous edges we chose a sign with even probability and set *β*_*st*_ = *w*_*st*_ or *β*_*st*_ = − *w*_*st*_. To ensure the *β* matrix is positive definite, we set the diagonal elements the largest value of the sum of the absolute value of the edge weights over each node. For continuous-discrete edges, in the linear dataset we set *ρ*_*st*_ = [−*w*, −.5*w*, .5*w*, *w*] and in the non-linear data we set *ρ*_*st*_ = *perm*([−*w*, −.5*w*, .5*w*, *w*]), where *perm* is a random permutation of the elements in the vector. For discrete-discrete edges we set the diagonal of *ϕ*_*rj*_(*y*_*r*_, *y*_*j*_) to *w*_*st*_ and the rest to -*w*_*st*_, while in the non-linear data we randomly set one parameter in each column and row to *w*_*st*_ and the rest to -*w*_*st.*_.

### Lung chronic disease data

The Lung Genomics Research Consortium (LGRC) contains multiple genomic datasets and clinical variables for two chronic lung diseases: chronic obstructive pulmonary disease (COPD) and interstitial lung disease (IDL). We used two data types from LGRC: gene expression profiles (15,261 probes) and clinical data for 457 patients (COPD *N* = 215; ILD *N* = 242). To expedite the execution time and avoid sample size problems, we only used the 530 most variant expression probes and 8 clinical variables: age, height, weight, forced expiratory volume in one second (FEV1), forced vital capacity (FVC), gender, cigarette history, and diagnosis (COPD or ILD). Age, height, weight and the spirometry variables (FEV1 and FVC) were divided into tertiles. Diagnosis was used for classification experiments.

### Graph estimation performance

Non-zero MGM edge parameters correspond to a prediction of the presence of that edge. For edges with multiple parameters, (i.e., *ρ*_*sj*_(*y*_*j*_) and *ϕ*_*rj*_(*y*_*r*_, *y*_*j*_)) if any of the parameters are non-zero we predict the edge is present. We use accuracy, precision and recall to evaluate edge recovery in our predicted graphs: precision is the ratio of true edge predictions to all edge predictions; recall is the ratio of true edge predictions to all edges in the true graph; accuracy is the ratio of true predictions to all predictions (in this case true prediction includes the correct predictions of the presence or absence of an edge); and the F1 score is the harmonic mean of precision and recall. In addition we consider the Matthews’ correlation coefficient (MCC) [20] which provides a correlation between the presence of edges in the true and predicted graphs. MCC is formulation of Pearson’s correlation for two binary variables so values of 1 correspond to perfect agreement between the variables, −1 to all disagreements, and 0 to random guessing. This measure is robust to unbalanced nature of the problem where in the true, sparse graph edge absence is much more frequent than edge presence.

### Functional enrichment and classification

For evaluation of the performance of various MGMs and other models on real data we used functional enrichment analysis of external databases and classification analysis over specific variables in the network, including disease diagnosis (for clinical datasets).

Gene annotations were retrieved from the Gene Ontology (GO) database [21] and we used the hypergeometric test to determine if sets of selected genes were overrepresented for any of these annotations (i.e., more occurrences of a given annotation were observed than we would expect from randomly selected genes).

Given the parameters learned from training data, ${\widehat{\varTheta}}_{train}$, we make predictions on any categorical variable, *y*_*target*_, in a testing dataset given the rest of the variables by selecting the category minimizes the negative log pseudolikelihood of the test data given the trained model:

$$ {\widehat{y}}_{target}= argmi{n}_{L_{target}}\ \tilde{l}\ \left({\widehat{\varTheta}}_{train};{x}_{test},\ {y}_{test\backslash target},\ {y}_{target}={L}_{target}\right) $$

We use this approach to predict lung disease diagnosis in a test dataset with an MGM trained with a training dataset.

We used 8-fold cross validation to determine the optimal classification settings of *λ* for MGM and Lasso, and which kernel to use for support vector machines (SVMs). We used the built-in Matlab implementations of Lasso and SVMs for these experiments.

## Results

### Synthetic data

#### Separate sparsities versus single sparsity parameter

**Fig. 1:**
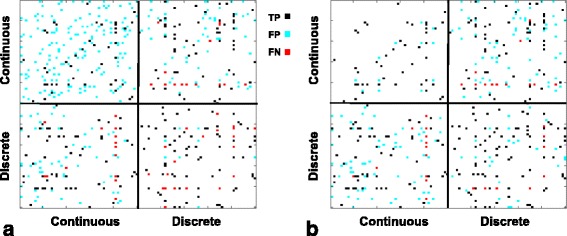
Example adjacency matrices predicted by an MGM, with sparsity selected using the oracle. **a** Single sparsity penalty *λ* = .19 **b** Split sparsity penalties *λ*_*cc*_ = .64, *λ*_*cd*_ = .19, *λ*_*dd*_ = .13

We applied Lee and Hastie’s method for learning an MGM to datasets simulated from a scale-free network. Initial experiments found that using a single sparsity penalty for all edge types produced many false positive continuous-continuous edge predictions, while missing many true discrete-discrete edges. We first present an example of this behavior on a single dataset of 500 samples over 50 four-level discrete variables and 50 continuous variables generated from a scale free network structure. Figure 1a shows the adjacency predictions of the learned MGM compared to the true graph using a *λ* selected by the oracle to minimize the number of edges present in one graph but not the other. This observation leads us to introduce separate sparsity penalties for each edge type. Figure 1b shows the adjacencies learned by an MGM with separate sparsity penalties for each edge type. For the sparsity parameters, the oracle searched over a range of 13 values evenly spaced on a log scale from .08 to .64.

**Fig. 2:**
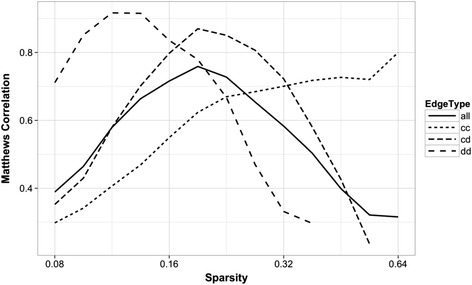
Matthews correlation between edge predictions and the true graph versus sparsity for the dataset from Fig. 1. Calculated for each edge type, cc for continuous-continuous, cd for continuous-discrete, dd for discrete-discrete, and over all edge predictions

Figure 2 shows the Matthews correlation of the edge predictions over the range of sparsity parameters, both overall and separated by edge type. For this example dataset, edge recovery of discrete-discrete edges had the highest MCC at *λ* = .13 while correlation of recovery of continuous-discrete edges was maximized at *λ* = .19 and continuous-continuous edges at *λ* = .64.

Selecting an optimal value for a single *λ* can be challenging, and the addition of two more sparsity parameters made it necessary to develop an efficient selection strategy. Other methods with multiple sparsity parameters search over a grid of models learned on all possible combinations of the parameters [22], but for our model the complexity of this selection would be cubic in the number of parameter values tested. Many model selection methods rely on calculating some likelihood over the training data, and it is not clear how to divide up this calculation by edge type. We do expect the presence of edges to remain relatively constant for a given edge sparsity parameter setting, so we extended a recent subsampling technique for model selection, StARS [1], to select three edge-type specific sparsity penalties by assuming independence between edge types. This assumption allows for a linear rather than cubic search over possible sparsity parameters. Thus, our method, StEPS, selects three sparsity penalties for Lee and Hastie’s MGM learning using a modified StARS approach for subsampling over different edge types.

#### StEPS outperforms other methods for model selection

Table 1 Summarizes graph prediction results for MGMs trained using sparsity penalties chosen with different model selection procedures over the 20 simulated non-linear datasets. Oracle, AIC, BIC, and CV evaluated models over a three dimensional grid of all possible combinations of *λ*_*cc*_, *λ*_*cd*_, *λ*_*dd*_ ∈ {.64, .32, .16, .08, .04}. For StARS, models were trained using a single sparsity penalty over the same range of values, and then either a single *λ* was selected based on the average instability over all edges or *λ*_*cc*_, *λ*_*cd*_ and *λ*_*dd*_ were selected based on the average instability of each edge type.

**Table 1:** Comparison of model selection methods

Methods	Precision	Recall	F1-score	Matthews CC	Accuracy
AIC	0.1104 (0.002)	0.9698 (0.004)	0.1982 (0.003)	0.2882 (0.003)	0.7952 (0.003)
BIC	0.4588 (0.028)	0.8633 (0.007)	0.5890 (0.025)	0.6098 (0.022)	0.9652 (0.004)
CV	0.1530 (0.003)	0.9694 (0.004)	0.2640 (0.005)	0.3539 (0.004)	0.8587 (0.003)
Oracle	0.9149 (0.015)	0.7868 (0.021)	0.8397 (0.009)	0.8416 (0.008)	0.9923 (0.000)
StARS – 1 *λ*	0.8988 (0.018)	0.4993 (0.010)	0.6408 (0.011)	0.6632 (0.011)	0.9854 (0.001)
StEPS – 3 *λ*	0.9159 (0.014)	0.6720 (0.009)	0.7731 (0.007)	0.7787 (0.007)	0.9897 (0.000)

*AIC* akaike information criterion, *BIC* Bayesian information criterion, *CV* cross-validation, Oracle: best possible prediction performance (maximize accuracy using true graph)

Mean (and standard error) of classification performance over 20 datasets simulated from scale-free networks

Our results show that AIC, BIC and CV produce overly dense models in the high-dimensional setting. Even when restricted to the single sparsity model, StARS significantly outperforms these traditional model selection methods. These results agree with what Liu et al. observed in their model selection experiments with the graphical lasso [1]. In addition, our modification of StARS with separate sparsities outperforms StARS with a single sparsity. Neither StARS model selection with 3 penalties nor the oracle model selection output a model where all three sparsities were equal in any of these experiments. Both methods always set *λ*_*dd*_ = .16 while the other parameters were always in the set { .64, .32, .16}. These results confirm the effectiveness of separating the MGM sparsity penalty into three *λ* values.

The original StARS procedure uses a subsampled dataset to make final edge predictions because the instability calculations are made on subsamples. We found, however, that in all cases the final edge prediction performance is higher if we use all samples compared to predictions from a model using a subsampled dataset. This improved performance is observed for all three metrics: accuracy, MCC, and F1. So, for all results presented below we used all samples to learn the MGM and make edge predictions with StARS selected sparsities.

It is important to note that because our method, StEPS, selects each sparsity parameter independently, so it incorrectly assumes that the instability of each edge type is independent of parameters of the rest. Without this assumption, we would have to perform stability experiments on all combinations of the sparsity parameters. To test if this assumption is reducing the edge recovery performance of StEPS, we ran StARS on the non-linear datasets using all 125 possible settings of λ_cc_, λ_cd_, λ_dd_ ∈ {.64, .45, .32, .23, .16}. This search space was chosen because all of the values selected by the Oracle or either of the other StARS methods fell in the set {.64,.32,.16} and additional intermediate values were needed to compare the relative performance of these methods. This experiment posed a new problem of how to monotonize and select the total instability over three dimensions rather than one. In addition, this experiment showed that the number of predicted edges in the graph does not always increase when one of the *λ* parameters decreases, even when the other two are held constant. We found that simply choosing the model with monotonized total instability closest to the user-specified *γ* threshold produced poor results. Taking into account the number of edges predicted across all subsamples for each parameter setting, as described below, was essential to producing usable results.

We fist looked at the total instability of the whole graph with all edge types pooled together, ${\widehat{D}}_{all}\left({\uplambda}_{\mathrm{cc}},{\uplambda}_{\mathrm{cd}},{\uplambda}_{\mathrm{dd}}\right)$. We monotonized this 3-dimensional matrix across each dimension: ${\overline{D}}_{all}\left({\uplambda}_{\mathrm{cc}},{\uplambda}_{\mathrm{cd}},{\uplambda}_{\mathrm{dd}}\right)\kern0.5em =\kern0.5em { \sup}_{\uplambda_{\mathrm{cc}},{\uplambda}_{\mathrm{cd}},{\uplambda}_{\mathrm{dd}}\le {t}_1,\ {t}_2,{t}_3}\ \widehat{D}\left({t}_1,\ {t}_2,{t}_3\right)$ and selected the setting of λ_cc_, λ_cd_, λ_dd_ that produced subsampled networks with the most edges such that ${\overline{D}}_{all}\left({\uplambda}_{\mathrm{cc}},{\uplambda}_{\mathrm{cd}},{\uplambda}_{\mathrm{dd}}\right)\le \gamma = .05$. Surprisingly, this approach performed worse than StEPS on all measures. MCC, for example, was significantly worse (mean of .845 for the heuristic versus .718 for this method, *t*-test *p* = 1.4e-4). We found that the networks produced by this method were too dense in the continuous-continuous edges and too sparse in continuous-discrete edges (results not shown). This is the result of averaging the instability of all edge types: the selected models were too stable for some edge types and too unstable for other types. To fix this, we separated the instability as before into ${\widehat{D}}_{cc}\left({\uplambda}_{\mathrm{cc}},{\uplambda}_{\mathrm{cd}},{\uplambda}_{\mathrm{dd}}\right)$, ${\widehat{D}}_{cd}\left({\uplambda}_{\mathrm{cc}},{\uplambda}_{\mathrm{cd}},{\uplambda}_{\mathrm{dd}}\right)$ and ${\widehat{D}}_{dd}\left({\uplambda}_{\mathrm{cc}},{\uplambda}_{\mathrm{cd}},{\uplambda}_{\mathrm{dd}}\right)$, and monotonized as before. Then we choose λ_cc_, λ_cd_, λ_dd_ that produced networks with the most edges such that $max\left({\overline{D}}_{cc}\left({\uplambda}_{\mathrm{cc}},{\uplambda}_{\mathrm{cd}},{\uplambda}_{\mathrm{dd}}\right),\ {\overline{D}}_{cd}\left({\uplambda}_{\mathrm{cc}},{\uplambda}_{\mathrm{cd}},{\uplambda}_{\mathrm{dd}}\right),\ {\overline{D}}_{dd}\left({\uplambda}_{\mathrm{cc}},{\uplambda}_{\mathrm{cd}},{\uplambda}_{\mathrm{dd}}\right)\right)\le \gamma = .05$. On 17 of the 20 datasets tested, this approach selected the same sparsity parameters as our proposed linear parameter search method. For the three runs where the two methods selected different parameters, the cubic search made better choices than the heuristic. Averaging over all runs the cubic search performed better than the heuristic but these results are not significant (e.g., mean MCC for the cubic search was .850 versus .845 for StEPS, *p* = 0.56). These results indicate that the independence assumption made by our heuristic is reasonable and that StEPS performs only slightly worse than a more theoretically sound cubic search while requiring much less computation.

#### Comparison to SCGGM

An important potential application of MGMs is in identifying expression quantitative trait loci (eQTLs) based on the predicted dependencies between single nucleotide polymorphisms (SNPs) and mRNA expression. The sparse conditional Gaussian graphical model (SCGGM) [22] is a method that addresses this problem specifically. Like many methods for finding eQTLs, the SCGGM assumes a linear relationship between the number of variant alleles and the mRNA expression level. Thus, the SCGGM is not technically a mixed graphical model because it treats the SNP allele counts as continuous variables. Another difference is that SCGGM does not predict discrete-discrete edges, which is also common among methods for finding eQTLs. Like StEPS, SCGGM also adopts a strategy of using a separate sparsity penalty for each edge type. SCGGM uses cross-validation to search over a two dimensional grid of parameter values in order to optimize prediction of continuous values given the discrete values.

**Fig. 3:**
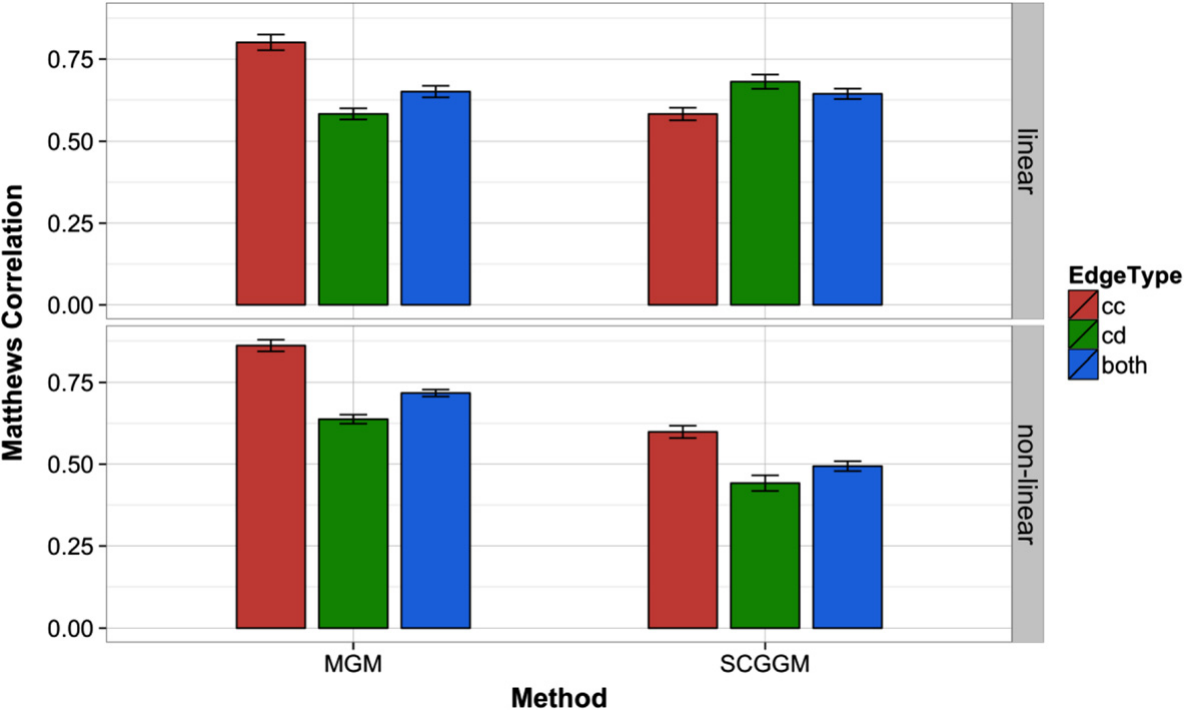
Comparison of edge recovery performance of MGM and SCGGM on continuous-continuous (cc), continuous-discrete (cd) and both edge types. Matthews correlation is averaged over 20 simulated datasets with linear continuous-discrete interactions and 20 datasets with non-linear interactions with error bars ± one standard error. Sparsity parameters for both methods selected by StEPS

First, we examined how our stability method can be used in SCGGM parameter selection instead of cross validation on our synthetic data and we found that StEPS resulted in significantly higher MCC (*p* < .01) for recovery of both continuous-continuous and continuous-discrete edge types. To perform a comparison between MGM and SCGGM edge predictions we used two sets of 20 mixed datasets generated from the same set of 20 scale-free networks but with parameters that resulted in either linear or non-linear interactions between discrete and continuous variables. Figure 3 shows the results of this experiment with StEPS selected sparsity parameters. As expected, MGM learning performed similarly on the linear and non-linear datasets because it does not assume linearity. The SCGGM had similar performance on continuous-continuous edge recovery with both datasets, but significantly worse performance on continuous-discrete edge recovery in the data with non-linear *cd* interactions, which resulted in worse overall performance in that setting.

For these tests we found that when allowing the selection of (different) edge type specific sparsity penalties, SCGGM chose the same penalty for the *cc* and *cd* edges in 36 out of the 40 datasets; and StEPS chose the same penalty for the *cc* and *cd* edges in 38 out of the 40 datasets, but a different *dd* penalty in all 40 cases.

### Performance of MGM on lung disease data

It is difficult to evaluate the edge recovery performance of MGM in real clinical datasets since the ground truth (all associations between variables) is not generally known. Alternatively, we evaluate MGM performance indirectly, by (1) recovering the small number of interactions that are known, (2) using external datasets (GO categories) to see if connected genes have similar function, (3) performing classification on a target variable in the network (disease diagnosis).

We applied our MGM learning approach to the LGRC biomedical data (described above). On this data StEPS selected the same value of *λ*_*cc*_, *λ*_*cd*_ = .2 for an average instability threshold of *γ* = .05 and *λ*_*cc*_, *λ*_*cd*_ = .1 for *γ* = .1. The selection of *λ*_*dd*_ proved more problematic. Even with *γ* = .1, *λ*_*dd*_ was selected to be so high that only one edge was selected (FEV1-FVC). This issue is likely caused by the fact that there are only 28 possible edges between the 8 clinical variables, and we expect that many of these variables are connected. Because of this and the fact that the experiments we perform below depend more on the continuous-discrete edges, we set all three penalties to the same value for our parameter searches in this section.

#### Recovering known interactions

**Fig. 4:**
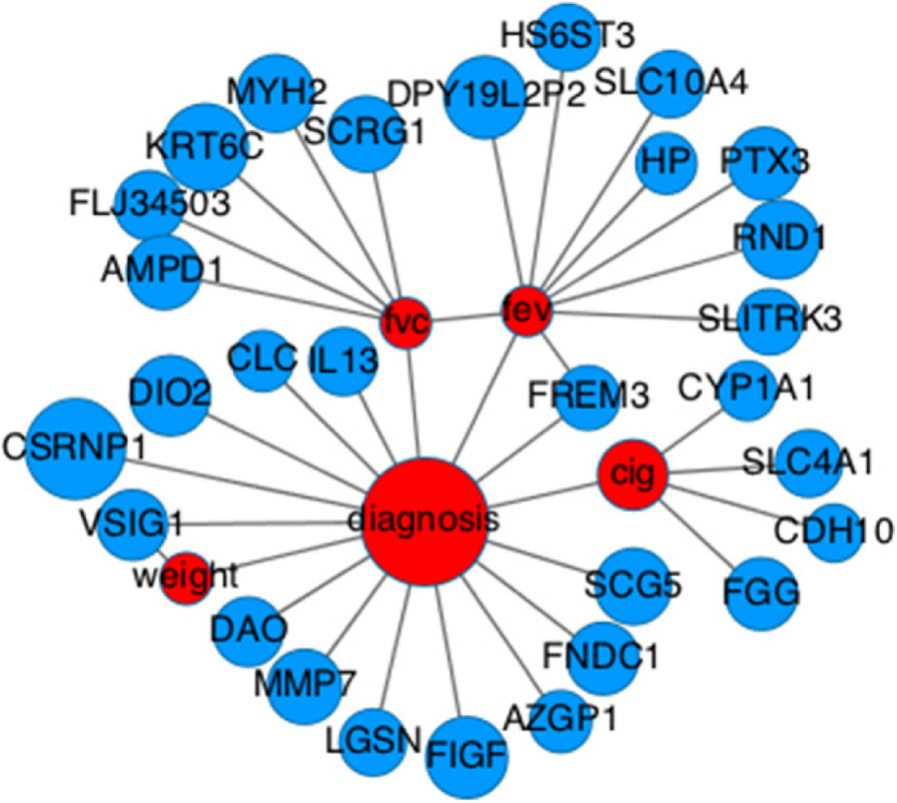
Learned sub-network of gene expression and clinical features connected to lung disease diagnosis, lung tests and cigarette smoking. Nodes are colored by data type, blue for gene expression, red for clinical variables. Edges were filtered by weight with a threshold of .05. Node size is proportional to the diagonal of the *β* matrix for continuous variables and *ϕ*_*yyF*_ for each categorical variable, y

Figure 4 shows part of the network learned over the lung (LGRC) dataset with *λ*_*cc*_, *λ*_*cd*_, *λ*_*dd*_ = .1. We only show the nodes adjacent to the clinical variables most relevant for lung disease: diagnosis, spirometry tests and cigarette smoking are shown in this graph. This model found a very strong connection between the FEV and FVC variables. A number of relevant gene expression variables are linked to diagnosis in this network. IL13 is part of the family of interleukin signaling molecules, which are associated with inflammatory response to tissue damage, and COPD is an inflammatory disease. We also see a link between diagnosis and MMP7, a previously discovered biomarker for idiopathic pulmonary fibrosis which is categorized as ILD [23]. A link between diagnosis and AZGP1, another previously studied marker for COPD [25], was also recovered. FGG and CYP1A1 were found to be linked to cigarette smoking history. CYP1A1 is known to convert polycyclic aromatic hydrocarbons, found in cigarette smoke, into carcinogens [26], and FGG codes for fibrinogen, a marker for inflammation, which is positively correlated with risk of mortality and COPD severity [24].

#### Recovering functional relationships

**Fig. 5:**
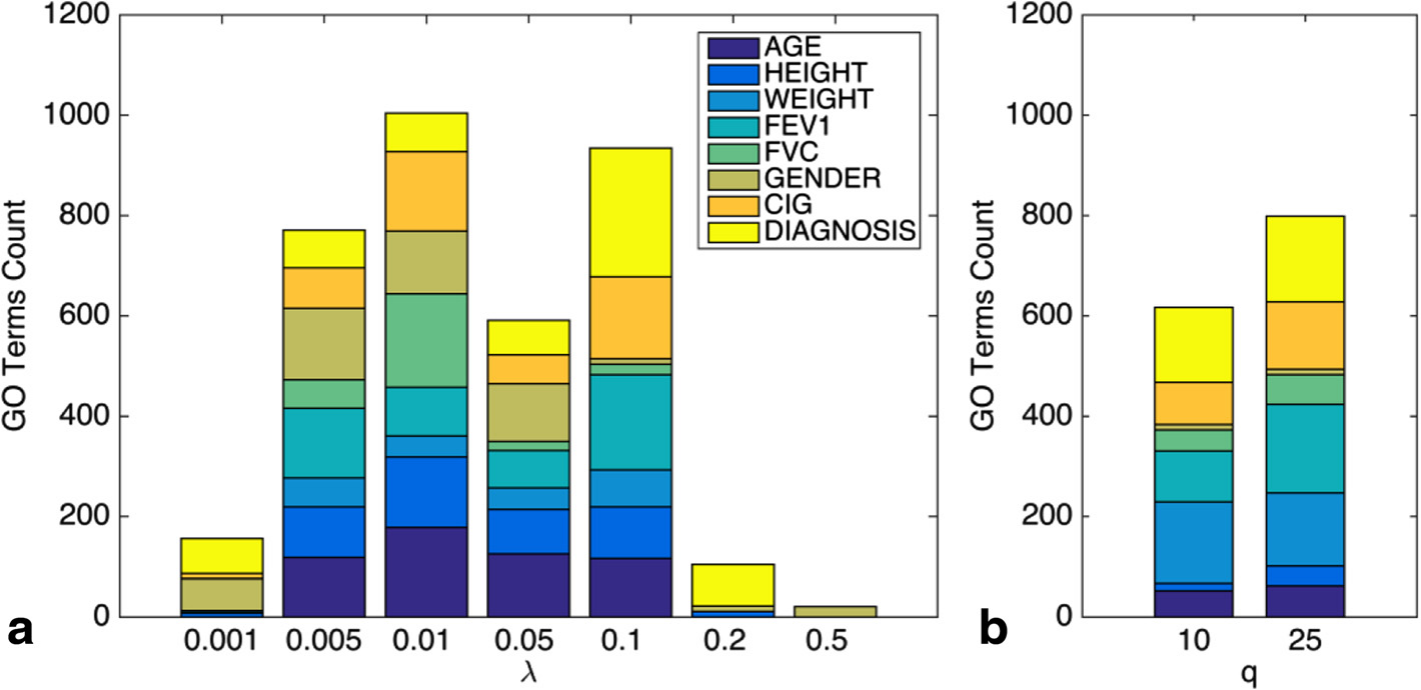
Counts of GO terms with uncorrected *p* < .05 for groups of genes with expression variables linked to each discrete clinical variable in **a** MGM networks at different values of λ and **b** qp-graphs at different values of q. Edge thresholds for qp-graphs were chosen to select similar numbers of connected genes to an MGM network with λ = .1

We also compared the functional relevance of MGM networks learned with StEPS and those learned by qp-graphs [27] which is another method for learning networks over mixed data. Like SCGGM, qp-graphs do not attempt to learn edges between two discrete variables, but qp-graphs do not make a linearity assumption about the discrete variables. To assess the biological relevance of networks learned at different levels of sparsity, we performed enrichment analyses on genes with expression variables linked to each clinical variable. For each group of genes linked to each clinical variable we counted GO terms with an uncorrected enrichment *p* < .05. These counts are shown in Fig. 5. Since each clinical variable represents a phenotype, we would hope that genes linked to those variables share similar biological function as measured by functional enrichment. We would like to choose a value of λ that maximizes the number of enriched GO terms.

The setting of λ = .1 recovers the most annotations for diagnosis, FEV1 and FVC, and also corresponds to an instability threshold of *γ* = .1. qp-graphs output a “non-rejection rate” for each edge, which corresponds to the number of different conditional independence tests that rejected the presence of each edge. To predict edges, this output needed to be thresholded, so we chose thresholds that produced similar numbers of edge predictions to λ = .1. While qp-graphs perform comparably well to MGMs in this test, we found the learning procedure to be very computationally expensive. On a quad-core laptop, learning a qp-graph with q = 25 took over 3 h (running time scales linearly with q) while learning an MGM took 4.4 min on average when the iteration limit was reached.

#### Evaluating MGM in classification tasks

**Fig. 6:**
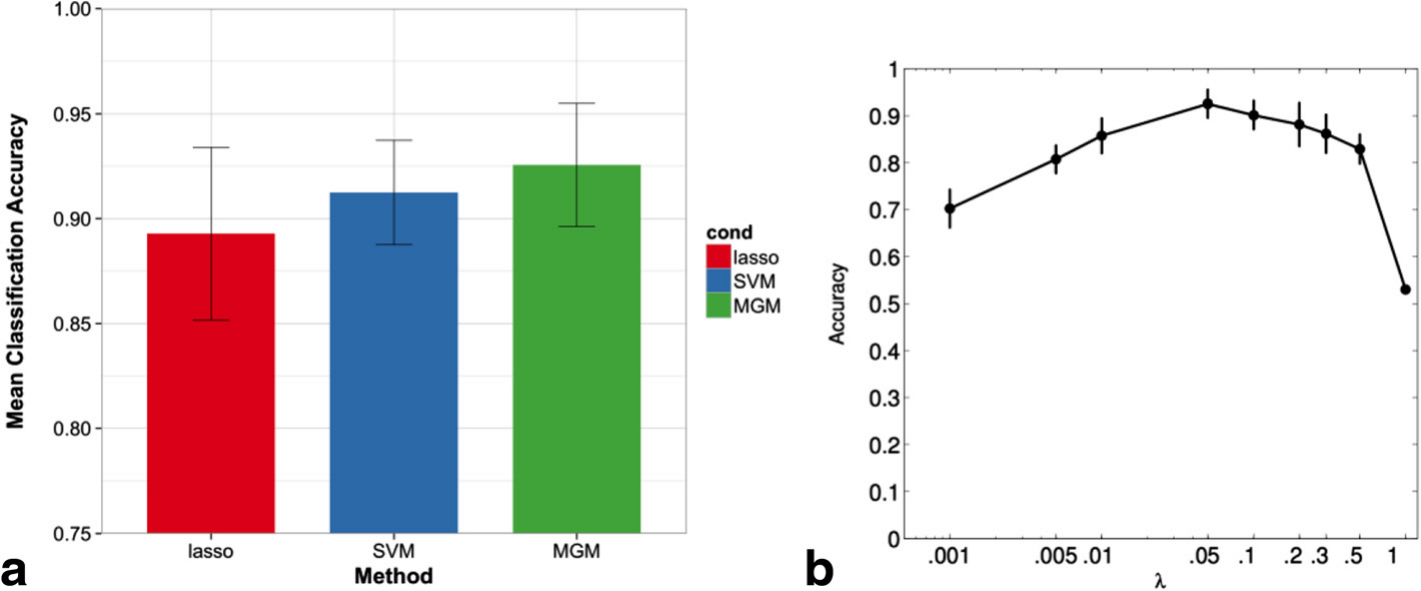
**a** 8-fold cross validation accuracies for COPD/ILD classification using different methods **b** Regularization effects on classification accuracy (with error bars of 1 standard deviation)

We also evaluated MGMs on how well a trained model can predict the status of a given target variable and we chose the lung disease diagnosis as a clinically relevant target variable. The MGM was compared to SVM and lasso. We optimized the settings of SVM, lasso and our mixed models to maximize the 8-fold cross-validation accuracy of predicting lung disease diagnosis using the 530 expression variables and 7 clinical variables. For SVM, we found that a linear kernel worked best on this data. For lasso and MGM, the parameter scan found that λ = 0.05 maximized this accuracy. Figure 6a shows a comparison between the optimized classification accuracies of these three methods. For MGM classification, we expected similar results to lasso because the conditional distribution of a discrete variable in the mixed model reduces to a (multivariate) logistic regression. It is interesting to see that the generative MGMs are not significantly different from discriminative lasso and SVM models in this experiment. While *λ* = .05 maximized the cross-validation accuracy for MGMs, Fig. 6b shows that the StARS selected sparsity values of *λ* = .1 and .2 do not perform significantly worse than *λ* = .05. Ιn addition, we ran experiments using StEPS with settings of [*λ*_*cc*_, *λ*_*cd*_, *λ*_*dd*_] = [.1, .1, .2] and [*λ*_*cc*_, *λ*_*cd*_, *λ*_*dd*_] = [.2, .2, .3] which correspond to instability thresholds of *γ* = .1 and *γ* = .05, respectively, and found that these changes did not significantly alter classification performance.

## Discussion

Learning graphical models over variables of mixed type is very important for biomedical research. The most widely used types of genomic data include continuous (gene expression, methylation, and protein data) and discrete (polymorphism and mutation data) variables. Similarly, clinical variables can be either continuous or discrete (numerical, categorical, boolean). We are interested in learning graphical models from these heterogeneous data to identify significant interactions between variables and uncover important biological pathways. As an added advantage, a learned network and joint probability can be used to ask an arbitrary number of classification questions over the data without the need for retraining each time [28]. These models would be broadly applicable to biological network inference, biomarker selection and patient stratification. Although calculating the MGM requires certain distributional assumptions about the data, the two distributions that make up the model in this work, a multivariate Gaussian for the continuous variables and a pairwise Markov random field for discrete variables, are well studied and have been successfully applied to many types of data. Additionally, using Gaussian copula methods [29] in conjunction with MGM learning would allow users to relax the normality assumption for the continuous data.

Our simulation study strongly supports the need for separate sparsity penalty for each edge type when learning an MGM. In addition we show the effectiveness of our extension of the StARS procedure, StEPS, to select these penalty terms. By using instability estimates from the single sparsity parameter model to select parameters for the three parameter model, we are making the assumption that each edge type set is independent from the others. We showed that StEPS performance under this independence assumption is comparable to a stability selection procedure that does not make this assumption. The payoff for StEPS is that we can select three parameters in linear time (over the number of parameter values searched) rather than cubic time. StEPS is a general methodology, which can be applied to a variety of mixed distribution settings, and will be especially useful in problems with many different edge types.

One could argue that StEPS substitutes an arbitrary setting of *λ* for an arbitrary setting of the instability threshold, *γ*. As Liu et al. point out, *γ* has a more intuitive meaning than *λ*, and we feel that setting this threshold compares to the common practice of setting an arbitrary significance threshold for rejecting the null hypothesis. Our results from applying StEPS to MGMs highlights the fact that the same setting of *γ* applies well to all edge types while different, edge type specific settings of *λ* are required for accurate edge recovery. Although it is possible to set the sparsity parameters based on some prior knowledge of the expected number of edges in the network, the data driven methods we present here allow for wide application of MGMs to domains where such knowledge is not available.

Furthermore, we show that our approach to MGM learning is competitive with a state-of-the-art eQTL learning method, SCGGMs. Although SCGGMs run faster that our MGMs due to the fact that it treats all variables as continuous, we showed that MGMs have a clear advantage when the discrete variables have non-linear relationships with the continuous variables. The assumption of linearity is common in eQTL learning and it makes sense in the haploid yeast datasets (e.g., [30]) used in the SCGGM study. In more complex organisms, however, an MGM that can handle non-linear interactions may be necessary.

While we had difficulty setting the discrete-discrete edge penalty in the lung dataset, we were still able to show the utility of MGM based analysis on biological data. Also, results from our classification experiment were robust to variation in the setting of this parameter. We do not expect MGMs to perform better than standard classification methods because the latter minimize the error of the classification problem (predicting the target variable given the rest) directly. The pseudolikelihood optimization in the MGM, however, must take into account the relationships between all of the variables. Despite this handicap, our results show, however, that the MGM-based classification is comparable to standard methods while offering two key advantages: (1) the same trained MGM can be used to make predictions about any variable without additional learning, and (2) the graph structure allows us to look at the second neighbors of the target variable and beyond for possible functional significance.

## Conclusions

Mixed graphical models are becoming popular in the statistics and machine learning literature, and there is a lot of potential for their application to high dimensional biological data. We have broached that potential in this study. We showed that MGMs can accurately learn undirected graphical models over a mixture of discrete and continuous variables in a high dimensional setting. In addition, we showed that using a separate sparsity parameter for each edge type in a graph can significantly improve edge recovery performance. These separate parameters can account for the differences in both the difficulty of learning such an edge and differences in the sparsity of edge types in the true graph. Finally, we showed that stability based methods are well suited for model selection in this setting and that our method StEPS allow us to perform a search over the sparsity penalties in linear time.

### Declarations

Publication charges for this article were funded by the National Institutes of Health (NIH) under award number R01LM012087, U54HG008540 and U01HL108642 for PVB, and award T32 EB009403 for AJS and RMD. The content is solely the responsibility of the authors and does not necessarily represent the official views of the NIH.

This article has been published as part of BMC Bioinformatics Volume 17 Supplement 5, 2016: Selected articles from Statistical Methods for Omics Data Integration and Analysis 2014. The full contents of the supplement are available online at http://bmcbioinformatics.biomedcentral.com/articles/supplements/volume-17-supplement-5.

## Abbreviations

AIC: Aikike information criterion
BIC: Bayesian information criterion
CV: cross-validation
eQTL: expression quantitative trait loci
MCC: Mathews correlation coefficient
MGM: mixed graphical model
SCGGM: sparse conditional gaussian graphical model
StEPS: stable edge-specific penalty selection
SVM: support vector machine
